# Transcriptional Responses of *Sclerotinia sclerotiorum* to the Infection by SsHADV-1

**DOI:** 10.3390/jof7070493

**Published:** 2021-06-22

**Authors:** Zheng Qu, Yanping Fu, Yang Lin, Zhenzhen Zhao, Xuekun Zhang, Jiasen Cheng, Jiatao Xie, Tao Chen, Bo Li, Daohong Jiang

**Affiliations:** 1 State Key Laboratory of Agricultural Microbiology, Huazhong Agricultural University, Wuhan 430070, China; quzhengwf@163.com (Z.Q.); zhenzhenchn@gmail.com (Z.Z.); Zhangxk2459@163.com (X.Z.); jiasencheng@mail.hzau.edu.cn (J.C.); jiataoxie@mail.hzau.edu.cn (J.X.); taochen@mail.hzau.edu.cn (T.C.); boli@mail.hzau.edu.cn (B.L.); 2 Hubei Key Laboratory of Plant Pathology, Huazhong Agricultural University, Wuhan 430070, China; yanpingfu@mail.hzau.edu.cn (Y.F.); yanglin@mail.hzau.edu.cn (Y.L.); 3 Hubei Hongshan Laboratory, Wuhan 430070, China

**Keywords:** transcriptome, *Sclerotinia sclerotiorum*, *Sclerotinia sclerotiorum* hypovirulence associated DNA virus 1, mycoviruses

## Abstract

The infection by a single-stranded DNA virus, Sclerotinia sclerotiorum hypovirulence-associated DNA virus 1 (SsHADV-1), causes hypovirulence, a reduced growth rate, and other colony morphological changes in its host *Sclerotinia sclerotiorum* strain DT-8. However, the mechanisms of the decline are still unclear. Using digital RNA sequencing, a transcriptome analysis was conducted to elucidate the phenotype-related genes with expression changes in response to SsHADV-1 infection. A total of 3110 *S. sclerotiorum* differentially expressed genes (DEGs) were detected during SsHADV-1 infection, 1741 of which were up-regulated, and 1369 were down-regulated. The identified DEGs were involved in several important pathways. DNA replication, DNA damage response, carbohydrate and lipid metabolism, ribosomal assembly, and translation were the affected categories in *S. sclerotiorum* upon SsHADV-1 infection. Moreover, the infection of SsHADV-1 also suppressed the expression of antiviral RNA silencing and virulence factor genes. These results provide further detailed insights into the effects of SsHADV-1 infection on the whole genome transcription in *S. sclerotiorum*.

## 1. Introduction

Mycoviruses or fungal viruses are parasitic viruses in various filamentous fungi and yeasts [1]. Since the first mycovirus causing the dieback disease of *Agaricus bisporus* was discovered, mycoviruses have been detected in different kinds of fungi gradually [1,2]. The most known species of mycovirus had a double-stranded RNA (dsRNA) genome, and some have single-stranded RNA (ssRNA) or single-stranded DNA (ssDNA) genome [3,4]. Usually, mycoviruses do not affect the phenotype of their hosts, but some could cause beneficial or detrimental effects on their hosts [3,5]. For *Saccharomyces cerevisiae*, the infection of L-A helper virus and toxin-coding killer virus converts normal yeasts into killer yeasts which secrete killer toxin to suppress sensitive yeast strains [6,7]. The infection of Talaromyces marneffei partitivirus-1 (TmPV1) enhances the virulence of *T. marneffei* in mice [8]. Mycovirus-induced hypovirulence could be found in many important plant hemi-biotrophic and necrotrophic pathogenic fungi, including *Aspergillus* spp., *Alternaria alternata*, *Bipolaris maydis*, *Botryosphaeria dothidea*, *Botrytis* spp., *Cryphonectria parasitica*, *Colletotrichum* spp., *Diaporthe* spp., *Fusarium* spp., *Helicobasidium mompa*, *Helminthosporium victoriae*, *Heterobasidion annosum*, *Macrophomina phaseolina*, *Magnaporthe oryzae*, *Ophiostoma* spp., *Penicillium digitatum*, *Pestalotiopsis theae*, *Rosellinia necatrix*, *Rhizoctonia solani*, *Sclerotinia* spp. and so on [3,9,10,11,12,13,14,15,16,17,18,19], indicating hypovirulence-related mycoviruses as potential resources to control plant fungal diseases [4].

*Sclerotinia sclerotiorum* (Lib.) de Bary is an important ubiquitous necrotrophic pathogen that can infect over 600 plant species [20]. Sclerotinia stem rot (SSR), caused by *S. sclerotiorum*, is a major disease and causes devastating economic losses of rapeseed (*Brassica napus* L.) around the world [21,22]. Nowadays, the researches of the pathogenic mechanism of *S. sclerotiorum* mainly focus on the plant cell wall-degrading enzymes (PCWDE), virulence-related secretory proteins and oxalic acid (OA) [20]. OA is considered a crucial virulence factor by manipulating the host redox environment, inducing programmed cell death, detoxifying calcium, and mediating pH signaling [23]. For *S. sclerotiorum*, five key genes of OA metabolism and regulation have been identified, including three biosynthesis genes (the malate synthase gene *Ss-mls1*, the oxaloacetate acetylhydrolase gene *Ss-oah1*, and the carnitine acetyl transferase gene *Ss-Pth2*), one oxalate decarboxylase enzyme gene (*Ss-odc2*) and one Zinc finger transcription factor gene (*Ss-Pac1*) [24,25,26,27].

To control SSR, the utilization of mycoviruses is an environmentally friendly method that could reduce the amount of chemical fungicide applications [28]. To date, fifteen families of mycoviruses have been identified in *S. sclerotiorum*, namely, *Megabirnaviridae*, *Partitiviridae*, *Reoviridae*, *Botybirnavirus*, *Endornaviridae*, *Hypoviridae*, *Fusariviridae*, *Botourmiaviridae*, *Solemoviridae*, *Mitoviridae*, *Alphaflexiviridae*, *Tymoviridae*, *Deltaflexiviridae*, *Mymonaviridae,* and *Genomoviridae*, of which 10 mycoviral species could confer hypovirulence to *S. sclerotiorum* and were considered as potential biological control agents (BCAs), including Sclerotinia sclerotiorum partitivirus 1, Sclerotinia Sclerotiorum mycoreovirus 4, Sclerotinia sclerotiorum botybirnavirus 2, Sclerotinia sclerotiorum hypovirus 1, Sclerotinia sclerotiorum hypovirus 2 (SsHV2-L), Hubei sclerotinia RNA virus 1, Sclerotinia sclerotiorum mitovirus 1, Sclerotinia sclerotiorum debilitation-associated RNA virus, Sclerotinia sclerotiorum negative-stranded RNA virus 1, and Sclerotinia sclerotiorum hypovirulence-associated DNA virus 1 (SsHADV-1) [3,29,30,31,32,33,34,35].

SsHADV-1, the prototype virus in the *Genomoviridae* family, is the first fungal circular single-stranded DNA virus. SsHADV-1 confers hypovirulence, reduced growth rate, small sclerotia, and abnormal colony morphology to its host *S. sclerotiorum* strain DT-8 [36]. SsHADV-1 infects and recruits a mycophagous insect, *Lycoriella ingenua*, as a vector to transmit itself among the *S. sclerotiorum* virus-free strains on rapeseed plants [37]. Moreover, SsHADV-1 can also switch its host from a fungal pathogen into an endophyte in rapeseed, which is based on the down-regulation of *S. sclerotiorum* virulence-associated genes regulated by SsHADV-1 [38]. This indicates that strain DT-8 may be a useful BCA to control SSR. The bio-priming treatment of rapeseed with *S. sclerotiorum* strain DT-8 can effectively control SSR and increase the yield in the field [39]. However, the cause of the abnormal phenotype of *S. sclerotiorum* strain DT-8 is still unknown.

RNA sequencing (RNA-seq) has been proven useful to unravel biological phenomena, and is also used to study the influence of viruses infection on their hosts [4,40]. For mycovirus, the comparative transcriptomics analysis is a common strategy to show the different expressions of fungal genes between the virus-infected and virus-free strains, and the most researches are about the RNA virus-mediated hypovirulent strains, such as Aspergillus fumigatus chrysovirus 41362 (AfuCV41362)-infected *A. fumigatus,* Botryosphaeria dothidea chrysovirus 1 and Botryosphaeria dothidea partitivirus 1-infected *B.* *dothidea*, Cryphonectria hypovirus 1 (CHV1)-infected *C. parasitica*, Fusarium graminearum hypovirus or Fusarium graminearum virus-infected *F. graminearum*, Rosellinia necatrix megabirnavirus 1-infected *R. necatrix*, SsHV2-L-infected *S. sclerotiorum,* and so on. Those researches show that the infection of mycoviruses can influence many important biological processes of their host, including primary and secondary metabolism, transcriptional regulation, signal transduction, substances transport, virulence factor expression, and ribosome function. Moreover, the infection of mycovirus also can change the small RNA accumulation of the hosts [41,42,43,44,45,46]. However, in most cases, the exact nature of mycoviruses-modulated gene expression of their host fungi is still unknown [47].

Although RNA-seq is a powerful tool, the sequence-dependent bias and inaccuracy of PCR amplification become obstacles for further applications [48]. To solve this problem, by labeling each cDNA molecule with a unique molecular identifier (UMI) before PCR amplification step, digital RNA-seq is created [48,49]. Rather than counting the number of reads, RNA abundance of digital RNA-seq is measured based on the number of unique barcode sequences observed for a given cDNA sequence, which can improve the accuracy of RNA-seq data [49,50]. In this study, digital RNA-seq was used to study the differential gene expression profiles between the hypovirulent *S. sclerotiorum* strain DT-8 and virulent virus-free strain DT-8VF at the vegetative stage. The transcriptional analyses of *S. sclerotiorum* to the infection by SsHADV-1 will enhance our understanding on the molecular mechanisms of the virus-mediated hypovirulence of pathogenic fungi.

## 2. Materials and Methods

### 2.1. Fungal Material and Growth Conditions

*S. sclerotiorum* hypovirulent strain DT-8 carrying SsHADV-1 (CCTCC M 2019328) was isolated from a sclerotium formed on a diseased stem of rapeseed from Hunan Province, China. The virulent SsHADV-1-free strain, DT-8VF (VF means virus-free), was derived from strain DT-8 by hyphal-tip isolation [36]. Both strains were grown on potato dextrose agar (PDA, Becton, Dickinson and Company, Sparks, MD, USA) plates at 20 °C, and stored on PDA slants at 4 °C.

### 2.2. Sample Collection and RNA Extraction

The mycelia of strains DT-8 and DT-8VF growing on PDA plates for 3 or 2 days when they had the highest growth rates were used to extract total RNAs using TRIzol (Invitrogen, Carlsbad, CA, USA) [51]. Then, DNA digestion was carried out using DNaseI (New England Biolabs, Beverly, MA, USA). The RNA quality was determined by examining A260/A280 with a NanodropTM OneCspectrophotometer (Thermo Fisher Scientific, Waltham, MA, USA). RNA integrity was confirmed by 1.5% agarose gel electrophoresis. 

### 2.3. cDNA Library Preparation and Sequencing

Qualified RNAs were finally quantified by Qubit 3.0 with a Qubit^TM^ RNA Broad Range Assay kit (Thermo Fisher Scientific Inc., Waltham, MA, USA). An amount of 2 μg of total RNAs was used for stranded RNA sequencing library preparation using KC-DigitalTM Stranded mRNA Library Prep Kit for Illumina^®^ (Catalog NO. DR08502, Wuhan Seqhealth technology Co., Ltd., Wuhan, China) following the manufacturer’s instructions. The kit eliminates the duplication bias during PCR and sequencing steps by using a UMI of 8 random bases to label the pre-amplified cDNA molecules. The products corresponding to 200–500 bps were enriched, quantified, and finally sequenced on Hiseq X 10 sequencer (Illumina, San Diego, CA, USA).

### 2.4. RNA-Seq Data Analysis

Raw sequencing data were first filtered by Trimmomatic (version 0.36) [52], and the low-quality reads were discarded and the reads contaminated with adaptor sequences were trimmed. Clean reads were further treated with KC-UID (the official analysis software of Seqhealth technology Co., Ltd. used to process reads of the digital RNA-seq library, https://github.com/KC-UID/KC-UID, accessed on 24 March 2021) to eliminate the duplication bias introduced during library preparation and sequencing. In brief, clean reads were first clustered according to the UMI sequences, in which reads with the same UMI sequence were grouped into the same cluster. Reads in the same cluster were compared to each other by pairwise alignment, and then reads with sequence identity over 95% were extracted to a new sub-cluster. After all the sub-clusters were generated, multiple sequence alignments were performed to obtain a consensus sequence for each sub-cluster. After these steps, any errors and biases introduced by PCR amplification or sequencing were eliminated. 

De-duplicated consensus sequences were used for standard RNA-seq analysis. They were mapped to the reference genome of *S. sclerotiorum* strain 1980 UF-70 (Assembly ASM14694v2) [53] using Spliced Transcripts Alignment to a Reference (STAR) software (version 2.5.3a) with default parameters [54]. Reads mapped to the exon regions of each gene were counted by featureCounts [55]. The differentially expressed genes (DEGs) were identified using the edgeR package [56]. To avoid the noise signals from high-throughput sequencing, genes detected only in at least three biological replicates of one condition, and above the detection threshold of 1 count per million (CPM) [57], were used in this analysis. The read counts were normalized separately by the trimmed mean of *M* values (TMM) method, and the DEGs were filtered by a threshold of false discovery rate (FDR) < 0.05 and an absolute log 2 fold change (logFC) > 1 [58]. A principal component analysis (PCA) was performed on the expression data using the “prcomp” function of R (version R x64 3.5.0; R Core Team, Vienna, Austria). Genes were annotated based on the BLAST results (*E*-value < 10^−5^) against two public databases: the Kyoto Encyclopedia of Genes and Genomes (KEGG) (http://www.genome.jp/kegg/, accessed on 28 March 2021) and InterPro (http://www.ebi.ac.uk/interpro/, accessed on 17 June 2021). The functional annotation of gene ontology (GO) terms was analyzed by BLAST2GO [59]. GO enrichment analysis was performed using the Biological Directed acyclic graphs Gene Ontology (BiNGO) 3.0.3 tool [60] with FDR < 0.05, and we paid more attention to the GO terms which were the end nodes in the directed acyclic graphs constructed by BiNGO [61]. KEGG enrichment was conducted using TBtools software v1.068 [62], and the threshold was set as *p*-value < 0.05. 

### 2.5. The Detection of Oxalic Acid (OA)-Producing Ability of the Two Strains

OA is reported to be a crucial virulence factor for *S. sclerotiorum.* To detect the OA-producing ability of strains DT-8 and DT-8VF, we measured the cumulative production rate of OA, which was expressed as the milligrams of oxalate produced per gram of mycelial dry weight in potato dextrose broth (PDB). PDB (50 mL) in 200 mL flasks was inoculated with two 9 mm actively growing mycelial disks from PDA. Three replicate flasks were prepared for both the strains. Control flasks were inoculated with plain PDA plugs. Cultures were statically incubated for 3 days at 20 °C. Mycelia were removed by vacuum filtration through Whatman number 1 filter paper, and the mycelial dry weight was determined after drying at 60 °C for 2 days. The production of OA in PDB was quantified by using a reverse-phase high-performance liquid chromatography (HPLC) system (Agilent, model 1260, Waldbronn, Germany). Culture filtrates were filtered through 0.45 μm membrane filters and used in HPLC analysis. The amount of OA present in 20 μL of the sample was separated and determined using HPLC with a standard curve constructed with oxalic acid anhydrate (Sigma-Aldrich, St. Louis, MO, USA). The column used was the 250 mm × 46 mm Hypersil C-18 (5 μm particles) from Thermo Fisher Scientific, Inc (Waltham, MA, USA). The mobile phase consisted of 0.5 mM tetrabutylammonium hydrogen sulfate and 0.036 M potassium dihydrogen orthophosphate in Milli-Q water adjusted to pH 2.0 with sulfuric acid. Concentration determination was performed using UV detection at a wavelength of 210 nm. The data were tested using Student’s *t*-test (*p* = 0.05) by SPSS Statistics 19.0.0.

### 2.6. Quantitative Real-Time RT-PCR (qRT-PCR) Analysis

qRT-PCR analysis for validating the differential expression data was prepared independently under the same conditions described above. First-strand cDNA was synthesized with an oligo d(T) primer by using cDNA Synthesis SuperMix (TransGen Biotech, Beijing, China). The qRT-PCR was carried out in a CFX96 Real-Time PCR Detection System (Bio-Rad, Hercules, CA, USA) with iTaq universal SYBR Green super mix (Bio-Rad, Hercules, CA, USA). PCR amplification was performed under the following conditions: 95 °C for 3 min, followed by 55 cycles of 95 °C for 15 s, 56 °C for 15 s, and 72 °C for 20 s. Melt curve profiles were analyzed for each gene tested at the end of each PCR reaction. The ubiquitin gene of *S. sclerotiorum* (*SS1G_11035*) served as an internal reference gene [29]. Primers for the target genes were designed using Beacon Designer V7.92 and are listed in Table S7. 

## 3. Results

### 3.1. Overview of All RNA-Seq Data

**Figure 1:**
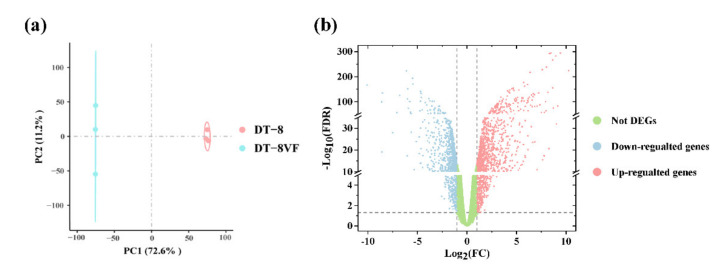
Transcriptome profile of digital RNA-Seq data. (**a**) The PCA for samples of strain DT-8 and strain DT-8VF. The blue and red ellipses display 95% confidence regions of samples of strain DT-8 and strain DT-8VF, respectively. (**b**) The volcano plot of digital RNA-seq data. A horizontal dotted line indicates significance cutoff and vertical lines indicate the differential expression magnitude cutoff.

For samples of strain DT-8 and strain DT-8VF, there were a total of 88 million and 59 million reads, of which an average of 90.30% and 95.63% reads were aligned to the *S. sclerotiorum* genome, respectively. Moreover, for libraries of strain DT-8, approximately 1.29% reads could be aligned to the SsHADV-1 genome while it was zero for strain DT-8VF (Table S1). According to the PCA, the three biological triplicates of each group clustered together (Figure 1a). A total of 9358 genes were detected above the detection threshold of 1 CPM in at least three biological replicates of one condition. In this study, the absolute logFC >1 and FDR < 0.05 were used to define DEGs. Compared to the gene expression data of strain DT-8VF, a total of 3110 statistically significant DEGs were found in strain DT-8 with 1741 up-regulated and 1369 down-regulated (Figure 1b).

For the 1741 up-regulated and 1369 down-regulated genes, 693 and 364 genes did not encode proteins with known domains according to the InterProScan. According to the frequency of occurrence of DEGs contained in each InterPro domain, InterPro domains were ranked and the 20 most abundant InterPro domains are shown in Table 1. For up-regulated genes, most of the hit InterPro domains were related to major facilitator superfamily (MFS) transporters (IPR036259: MFS transporter superfamily; IPR020846: major facilitator superfamily domain; IPR011701: major facilitator superfamily), including the sugar transporter (IPR005829; sugar transporter and conserved site; IPR003663: sugar/inositol transporter) and amino acid permeases (IPR002293: amino acid/polyamine transporter I). For the down-regulated genes, InterPro domains associated with RNAse H (IPR036397: Ribonuclease H superfamily; IPR012337: Ribonuclease H-like superfamily), an important component of antiviral RNA silencing [63], were detected. This suggests that the antiviral RNA silencing of strain DT-8 might be inhibited by SsHADV-1. Moreover, InterPro domains related to the fungal transcriptional regulatory proteins (IPR001138: Zn(2)-C6 fungal-type DNA-binding domain; IPR036864: Zn(2)-C6 fungal-type DNA-binding domain superfamily; IPR013087: Zinc finger C2H2-type) were also identified. These down-regulated genes of the fungal transcription factors might be associated with the reduced growth of strain DT-8.

### 3.2. Gene Ontology (GO) Enrichment Analysis for DEGs

**Figure 2:**
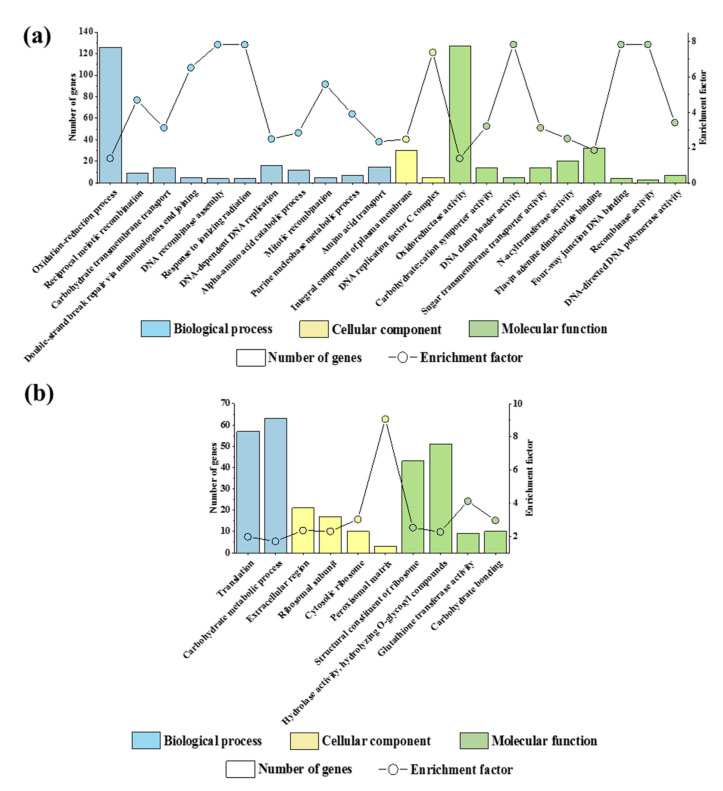
The GO enrichment analysis of DEGs. (**a**) The GO enrichment analysis of the up-regulated genes. (**b**) The GO enrichment analysis of the down-regulated genes.

For the 1741 up-regulated genes, there were 22 significantly enriched GO terms which were end nodes in the directed acyclic graphs constructed by BiNGO (Figure 2a, Table S2). Nine GO terms were related to the DNA replication and DNA repair, namely, “reciprocal meiotic recombination”, “DNA-dependent DNA replication”, “DNA recombinase assembly”, “double-strand break repair via nonhomologous end joining”, “mitotic recombination”, “purine nucleobase metabolic process”, “DNA replication factor C complex”, “DNA-directed DNA polymerase activity”, “four-way junction DNA binding”, and “DNA clamp loader activity”. Moreover, “carbohydrate transmembrane transport”, “carbohydrate: proton symporter activity”, and “sugar transmembrane transporter activity” were also significantly enriched. These results showed that the infection of SsHADV-1 might activate the DNA damage response and enhance the carbohydrate acquisition of strain DT-8.

The 1369 down-regulated genes were significantly enriched to 10 end node GO terms (Figure 2b, Table S3) and three significantly enriched GO terms were related to the structure and function of the ribosome, namely, “ribosomal subunit,” “cytosolic ribosome,” and “structural constituent of ribosome.” Meanwhile, “translation” was also the enriched GO term. There were also two GO terms associated with carbohydrate metabolism, namely, “carbohydrate binding” and “carbohydrate metabolic process.” These GO terms might be related to the reduced growth of strain DT-8. 

### 3.3. KEGG Enrichment Analysis of DEGs

**Figure 3:**
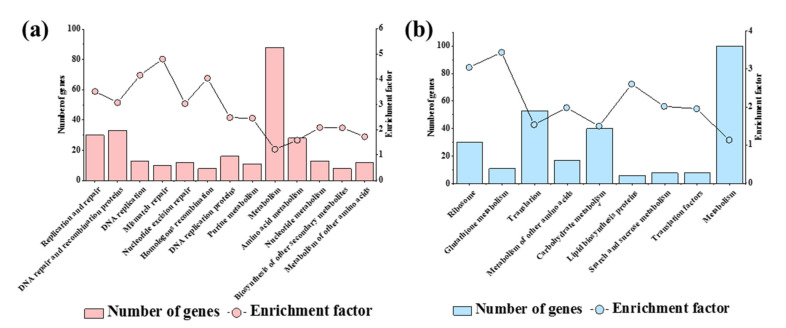
The KEGG enrichment analysis of DEGs. (**a**) The KEGG enrichment analysis of the up-regulated genes. (**b**) The KEGG enrichment analysis of the down-regulated genes.

The KEGG enrichment analysis of the up-regulated *S. sclerotiorum* genes showed similar results to the GO enrichment analysis. For the 1741 up-regulated genes, there were 12 significantly enriched pathways, of which 8 pathways were related to DNA replication and DNA repair, including “replication and repair”, “DNA repair and recombination proteins”, “DNA replication”, “mismatch repair”, “nucleotide excision repair”, “homologous recombination”, “DNA replication proteins” and “nucleotide metabolism” (Figure 3a, Table S4). These pathways also showed that the infection of SsHADV-1 might activate the DNA damage response of strain DT-8.

For the down-regulated genes, similar to the GO enrichment analysis, the “carbohydrate metabolism”, “starch and sucrose metabolism”, “ribosome”, “translation”, and “translation factors” were the enriched pathways. Moreover, the “lipid biosynthesis proteins” were also enriched (Figure 3b, Table S5). These pathways also might be related to the reduced growth of strain DT-8.

### 3.4. The Key Non-Homologous End Joining (NHEJ) Genes Were Up-Regulated in Strain DT-8

**Figure 4:**
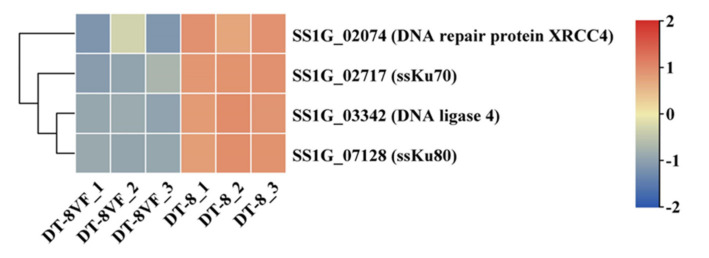
The expression profiles of the key NHEJ genes.

Ku70-Ku80 is a DNA-binding heterodimer that forms a complex with the DNA repair protein XRCC4 and the DNA ligase 4 to activate the NHEJ pathway for the repair of DNA double-strand breaks [64]. In the *S. sclerotiorum* genome, there are two Ku70/Ku80 homologs (ssKu70, SS1G_02717; ssKu80, SS1G_07128) [65], one DNA repair protein XRCC4 (SS1G_02074), and one DNA ligase 4 (SS1G_03342) (Table S6). Compared to those in strain DT-8VF, all the key NHEJ genes were up-regulated in strain DT-8 (Figure 4). This result suggested that the infection of SsHADV-1 activated the NHEJ pathway in strain DT-8.

### 3.5. Most of the Antiviral RNA Silencing Genes Were Down-Regulated in Strain DT-8

**Figure 5:**
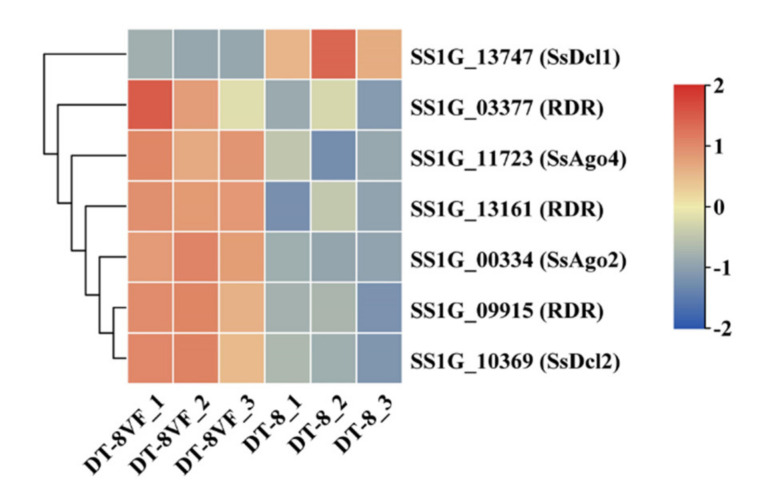
The expression profiles of antiviral RNA silencing genes.

RNA silencing is identified as an adaptive defense mechanism against foreign nucleic acids, including viruses in animals, fungi, and plants [66,67]. In the *S. sclerotiorum* genome, there were two Dicer-like (Dcl) genes, two argonaute-like (Agl) genes, and three RNA-dependent RNA polymerase (RDR) genes [46]. Compared to strain DT-8VF, except for the *SsSDcl1* (*SS1G_13747*), the other antiviral RNA silencing genes were down-regulated in strain DT-8 (Figure 5). It suggested the SsHADV-1 might suppress the antiviral RNA silencing to survive in strain DT-8.

### 3.6. SsHADV-1 Down-Regulated the Expression of Many Virulence Factor Genes

**Figure 6:**
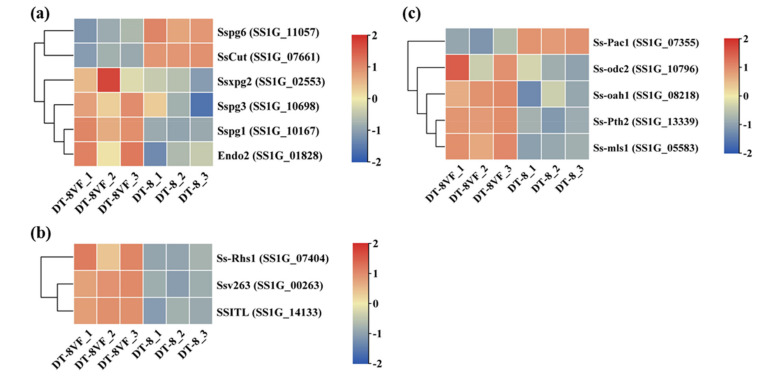
The expression profiles of *S. sclerotiorum* virulence factor genes. (**a**) The expression levels of PCWDE genes previously identified. (**b**) The expression levels of secretory protein encoding genes. (**c**) The expression levels of OA metabolism and regulation genes.

Among the previously identified genes of PCWDE and effector-like small secretory protein [68], *Sspg2*, *Sspg1*, *Sspg3*, *Endo2*, *Ssv263*, *SSITL*, and *Ss-rhs1* were down-regulated in strain DT-8 (Figure 6a,b). Compared to that in strain DT-8VF, except for the positive transcription factor gene *Ss-Pac1*, the expression of key genes of OA biosynthesis (*Ss-Oah1*, *Ss-Pth2*, and *Ss-Mls1*) and degradation (*Ss-odc2*) were also downregulated in strain DT-8 (Figure 6c). This showed that the infection of SsHADV-1 might comprehensively suppresses the OA metabolism of strain DT-8.

### 3.7. SsHADV-1 Did Not Influence the OA-Producing Ability

To evaluate the OA-producing ability between the two strains, we detected the cumulative production rate of OA. The cumulative production rates of OA of the two strains increased from the 1st to the 3rd day and were not significantly different (Figure S1). This showed that the SsHADV-1 infection did not influence the OA-producing ability of strain DT-8.

### 3.8. Gene Expression Level by qRT-PCR

To validate the results obtained in the digital RNA-seq experiments, qRT-PCR was used to analyze the relative expression levels of 12 *S. sclerotiorum* genes. The results showed the expression patterns of these representative genes were consistent with the transcriptome data (Figure S2), which indicated that the transcriptome data were reliable.

## 4. Discussion

In this research, we analyzed the gene expression of strain DT-8 compared to strain DT-8VF, and studied the effects of SsHADV-1 infection on the whole genome transcription in *S. sclerotiorum*. We found that the SsHADV-1 infection down-regulated the expression of genes involved in carbohydrate and lipid metabolism, ribosomal assembly, translation, and virulence factors. This might be associated with the reduced growth and hypovirulence of strain DT-8. Moreover, SsHADV-1 infection inhibited antiviral RNA silencing, and activated the DNA replication and DNA damage response processes in strain DT-8. Those DEGs might be the key factors through which SsHADV-1 could successfully parasitize and replicate in strain DT-8. 

Previously, Zhang et al. compared the gene expression between strains DT-8 and DT-8VF on rapeseed leaves and found that many important virulence-associated genes were down-regulated in strain DT-8 [38]. In this study, we also found SsHADV-1 down-regulated the expression of many virulence factor genes of strain DT-8 on PDA medium. In planta, there were 18 DEGs encoded PCWDE and secretory proteins, of which 2 up-regulated genes (*Sscut* and *Sspg6*) and 7 down-regulated genes (*Sspg2*, *Sspg1*, *Sspg3*, *Endo2*, *Ssv263*, *SSITL*, and *Ss-rhs1*) were common in vitro. According to KEGG enrichment analysis, both in vitro and in planta, the most enriched KEGG pathways of up-regulated genes were related to the DNA replication and DNA repair. For the down-regulated genes, the “starch and sucrose metabolism” was the only common KEGG pathway. In planta, many enriched KEGG pathways of down-regulated genes were associated with metabolism of secondary metabolites. This showed that infection of SsHADV-1 could stably affect some important *S. sclerotiorum* genes, but also could regulate expressions of different host genes in response to the changes of lifestyle, when strain DT-8 is grown in different environments.

OA has shown to influence the infection of *S. sclerotiorum* [20]. When strain DT-8 grew on rapeseed leaves, the expression of OA metabolic genes was not lower than strain DT-8VF [38]. This suggested that the OA might also have an important role in the colonization of strain DT-8 in rapeseed. In our study, the expression of both key OA biosynthesis and degradation genes of strain DT-8 was lower than that of strain DT-8VF. It is not surprising that OA-producing ability of strain DT-8 was not influenced. This is another example that OA is one of the virulence factors for *S. sclerotiorum*. 

The mycovirus-induced phenotype is partly due to metabolic changes induced by the viral infection [69]. As a fundamental biochemical process, carbohydrate metabolism ensures a constant supply of energy to living cells [70]. Various findings have showed that a virus infection could influence the carbohydrate metabolism of host fungi [42]. Lee Marzano et al. found the infection of SsHV2-L up-regulated the sugar transporter genes of *S. sclerotiorum* [46]. The gene ontology-like functional catalog (FunCat) analysis showed that the largest category of down-regulated genes in AfuCV41362-infected *A. fumigatus* was “C-compound and carbohydrate metabolism” [45]. In this study, we also found that a large number of up-regulated or down-regulated genes were enriched in carbohydrate transmembrane transport or carbohydrate metabolism pathways in strain DT-8. These results suggested that the infection of SsHADV-1 enhanced the carbohydrate acquisition of strain DT-8 but reduced carbohydrate metabolism. This might be a reason for the reduced growth of strain DT-8.

In eukaryotes, RNA silencing has been shown to function primarily in the defense against invasive nucleic acids, such as the infection of viruses [66]. In *Arabidopsis thaliana*, two DNA viruses, cabbage leaf curl virus and cauliflower mosaic virus, were targeted by all four *A. thaliana* DCLs [71]. For fungi, both CHV1 and Aspergillus virus 341 are the targets of their host RNA silencing machinery [72,73]. Meanwhile, viruses have evolved strategies to counteract the host RNA silencing responses, such as encoding RNA silencing suppressors (RSS). The RSS βC1 encoded by the satellite of plant DNA virus, tomato yellow leaf curl China virus, can up-regulate *Nicotiana benthamiana* calmodulin-like protein, which appears to be an endogenous suppressor of RNA silencing, to suppress RNA silencing through repressing the expression of RNA-dependent RNA polymerase 6 (RDR6) [74]. For mycoviruses, RSS is also an important strategy to suppress the RNA silencing of the host, such as CHV1 and Rosellinia necatrix mycoreovirus 3 [73,75]. For *S. sclerotiorum*, there is a robust RNA silencing mechanism with important roles in fungal antiviral defense, and *SsAgl2*, *SsDcl1*, and *SsDCl2* are key genes to defend against fungal RNA viruses or DNA viruses [76,77]. Through the digital RNA-seq data, we found that the infection of SsHADV-1 down-regulated most RNA silencing genes of strain DT-8. It might be important for SsHADV-1 to survive in strain DT-8. 

Viral DNA genomes have a limited coding capacity and therefore harness cellular factors of the host to generate progeny virions [78]. By hijacking and manipulating host DNA replication and DNA damage response processes, DNA viruses can selectively utilize or abrogate components of the cellular machinery to complete their life cycles [79]. The smaller the viral genome, the more minimal the coding capacity, and the greater the need to harness cellular processes of the host [80]. As a circular ssDNA mycovirus, the genome of SsHADV-1 is only 2166 nt, coding for one replication initiation protein (Rep) and one coat protein (CP) [36]. In our research, for the up-regulated genes, there were numerous enriched GO terms or KEGG pathways which were related to DNA replication and DNA damage response processes. This might be the embodiment in which SsHADV-1 utilized cellular processes of strain DT-8 to complete the replication. Moreover, we found that the key NHEJ genes (*ssKu70*, *ssKu80*, *SS1G_02074*, and *SS1G_03342*) were up-regulated in strain DT-8. These genes have been proven to be related to the replication of DNA virus. Choi et al. presented evidence both in vivo and in vitro that Ku70/80 stimulates the replication of the linear single-stranded DNA virus, adeno-associated virus, in the presence of both adenovirus and herpes simplex virus coinfection [81]. Muylaert and Elias found that the RNAi-mediated knockdown of DNA ligase IV and its co-factor XRCC4 caused a hundred-fold yield reduction of linear double-stranded DNA virus, Herpes simplex virus type I, in human 1BR.3.N fibroblasts [82]. For SsHADV-1, these key NHEJ genes might also be key factors for replication in strain DT-8.

## 5. Conclusions

Previously, we investigated the early transcriptional response when *S. sclerotiorum* hyphae were inoculated with purified SsHADV-1 virions. The results showed that SsHADV1 infection could influence the host Ras-small G protein signal transduction pathway, which might modulate changes in host metabolism to provide the energy for SsHADV-1 invasion and proliferation [29]. In this study, to further study the influence of SsHADV-1 infection on its fungal host, we performed digital RNA-seq and studied the different gene expression profiles between the hypovirulent strain DT-8 and virulent virus-free strain DT-8VF at the vegetative stage. We found the SsHADV-1 infection could influence carbohydrate metabolism, suppress the expression of some virulence factors and antiviral RNA silencing genes, and activate the DNA replication and DNA damage response processes. These results provide a view of expression difference of *S. sclerotiorum* genes between control and the infection of SsHADV-1, and the mechanisms underlying needs further study.

## Figures and Tables

**Table 1:** The most abundant InterPro domains according to the InterProScan.

Up-Regulated Genes			Down-Regulated Genes		
InterPro ID	Description	No. of Genes	InterPro ID	Description	No. of Genes
IPR036259	MFS transporter superfamily	73	IPR036291	NAD(P)-binding domain superfamily	50
IPR020846	Major facilitator superfamily domain	71	IPR027417	P-loop containing nucleoside triphosphate hydrolase	42
IPR027417	P-loop containing nucleoside triphosphate hydrolase	56	IPR017853	Glycoside hydrolase superfamily	34
IPR036291	NAD(P)-binding domain superfamily	49	IPR020846	Major facilitator superfamily domain	31
IPR011701	Major facilitator superfamily	41	IPR029058	Alpha/Beta hydrolase fold	31
IPR029058	Alpha/Beta hydrolase fold	33	IPR036259	MFS transporter superfamily	31
IPR036188	FAD/NAD(P)-binding domain superfamily	28	IPR036397	Ribonuclease H superfamily	25
IPR029063	S-adenosyl-L-methionine-dependent methyltransferase	23	IPR012337	Ribonuclease H-like superfamily	24
IPR016181	Acyl-CoA N-acyltransferase	22	IPR029063	S-adenosyl-L-methionine-dependent methyltransferase	24
IPR005828	Major facilitator, sugar transporter-like	20	IPR011701	Major facilitator superfamily	23
IPR002347	Short-chain dehydrogenase/reductase SDR	18	IPR002347	Short-chain dehydrogenase/reductase SDR	22
IPR005829	Sugar transporter, conserved site	18	IPR036188	FAD/NAD(P)-binding domain superfamily	21
IPR006600	HTH CenpB-type DNA-binding domain	18	IPR036396	Cytochrome P450 superfamily	21
IPR036396	Cytochrome P450 superfamily	18	IPR001128	Cytochrome P450	20
IPR001128	Cytochrome P450	17	IPR001138	Zn(2)-C6 fungal-type DNA-binding domain	19
IPR003593	AAA+ ATPase domain	17	IPR011009	Protein kinase-like domain superfamily	18
IPR000182	GNAT domain	16	IPR009057	Homeobox-like domain superfamily	17
IPR002293	Amino acid/polyamine transporter I	16	IPR036864	Zn(2)-C6 fungal-type DNA-binding domain superfamily	16
IPR003663	Sugar/inositol transporter	15	IPR000477	Reverse transcriptase domain	15
IPR011009	Protein kinase-like domain superfamily	15	IPR013087	Zinc finger C2H2-type	14
IPR017853	Glycoside hydrolase superfamily	15	IPR020904	Short-chain dehydrogenase/reductase, conserved site	14
			IPR036249	Thioredoxin-like superfamily	14

## Data Availability

All raw data of RNA-seq are available at Sequence Read Archive (PRJNA695466).

## Supplementary Materials

The following are available online at https://www.mdpi.com/article/10.3390/jof7070493/s1: Figure S1: The cumulative production rate of OA of strains DT-8 and DT-8VF; Figure S2: The expression of *S. sclerotiorum* genes detected by qRT-PCR and RNA-seq; Table S1: Summary of sequencing data. Table S2: The GO enrichment analysis of the up-regulated genes; Table S3: The GO enrichment analysis of the down-regulated genes; Table S4: The KEGG enrichment analysis of the up-regulated genes; Table S5: The KEGG enrichment analysis of the down-regulated genes; Table S6: The InterPro domains of *SS1G_03342* and *SS1G_02074*; Table S7: The qRT-PCR primers used in this study.
