# Supplementary material for: Transcriptional Responses of Sclerotinia sclerotiorum to the Infection by SsHADV-1
Source: J Fungi (Basel). 2021 Jun 22;7(7):493. doi: 10.3390/jof7070493 (PMC8303302; doi:10.3390/jof7070493)
Supplement: Supplementary file 1 [file jof-07-00493-s001.zip › ╕╜┬╝/Supplementary Figure S1.pdf]

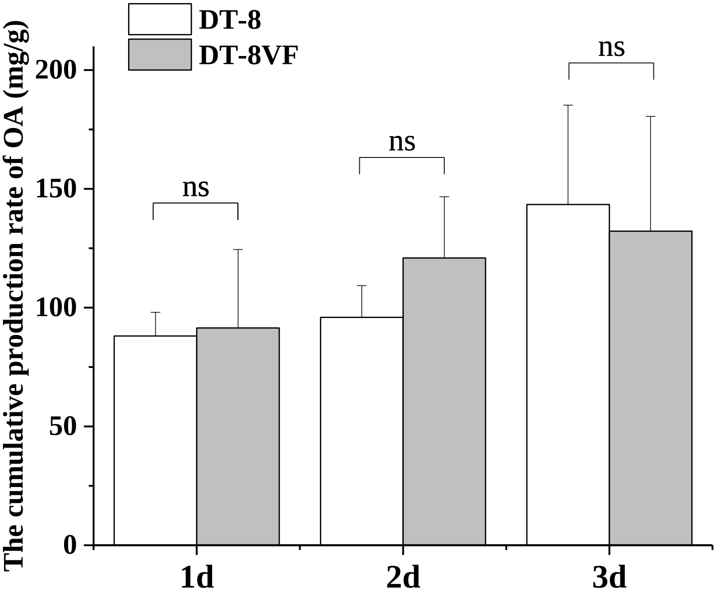

Figure S1 The cumulative production rate of OA of strain DT-8 and DT-8VF. Results are means of three replicates per sample including standard deviation. Data were analyzed by the Student's t-test (P=0.05). ns: non-significant differences.
