# Supplementary material for: Transcriptional Responses of Sclerotinia sclerotiorum to the Infection by SsHADV-1
Source: J Fungi (Basel). 2021 Jun 22;7(7):493. doi: 10.3390/jof7070493 (PMC8303302; doi:10.3390/jof7070493)
Supplement: Supplementary file 1 [file jof-07-00493-s001.zip › ╕╜┬╝/Supplementary Figure S2.pdf]

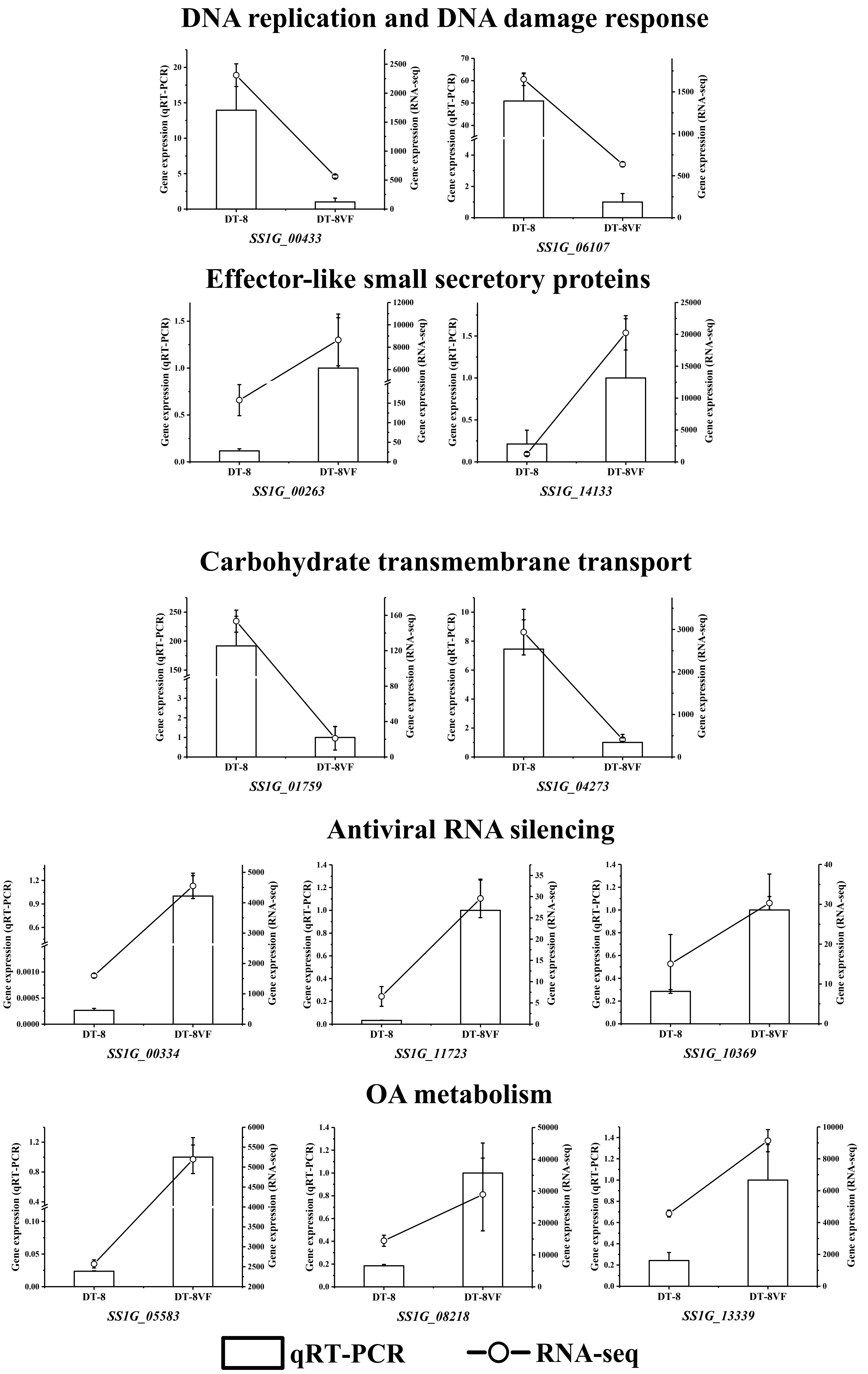

Figure S2 The expression of *S. sclerotiorum* genes detected by qRT-PCR and RNA-seq
